# Centenary of The Death of józef Brudziński: on His Contribution To Early Bacteriology

**DOI:** 10.34763/devperiodmed.20172103.293296

**Published:** 2017-10-28

**Authors:** Piotr Polaczek

**Affiliations:** 1 Braun Labs, California Institute of Technology, Pasadena, California U.S.A

**Fig. 1:**
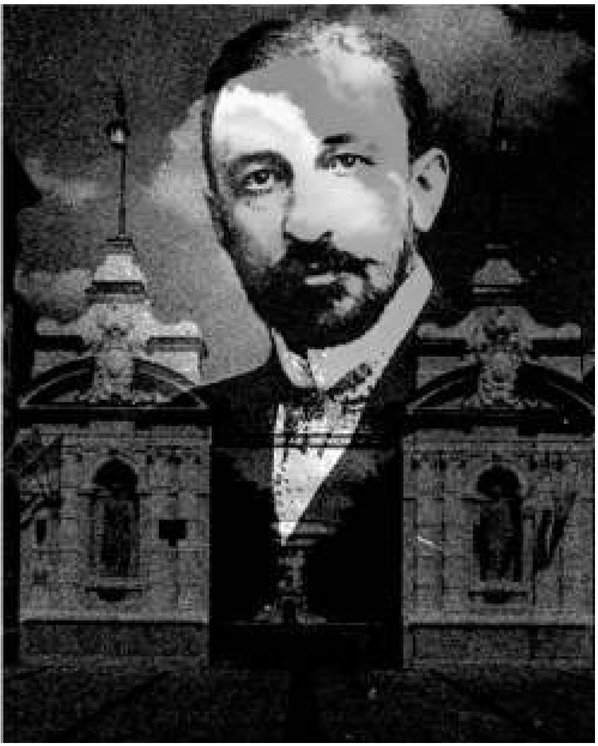
A photo of Józef Brudziński superimposed over the entrance gate to Warsaw University, where in 1916 he placed a white eagle, as a testament to the Polish character of the school.

Józef Brudziński died December 18^th^, 1917 at the age of 43. In his short life, he achieved prominence as a pediatrician, neurologist, bacteriologist, and also as a political figure. In Poland, he is remembered as the first rector of Warsaw University (fig. 1) following its revival after a century of turmoil (partition of Poland, several uprisings, and World War I). He was also wholeheartedly involved in organizing several pediatric hospitals in Poland, at the time among the most modern in Europe. His work as a pediatrician on neurological signs in diagnosis of meningitis is widely known. Medical students all over the world are familiar with the tongue-twister, called Brudziński’s sign or relex, used in the diagnosis of meningitis. Less is known about his contributions to bacteriology and his seminal work on intestinal bacteria. One has to dig deep into the literature of the early 20^th^ century to gain insight into this equally important work. In online searches, the name Brudziński complicates matters, as the spelling of his name has many variations in the literature: Brudzińsky, Brudzinske, Brudsinski etc. The approaching hundredth anniversary of his death is an appropriate time to call attention to his largely forgotten early work that, along with that of a handful of others pioneers, forms the foundation of what we now call probiotic and microbiome research.

Because in partitioned Poland, at the end of 19^th^ century there were limited opportunities to study medicine, Brudziński left the country and enrolled in the medical school programs in Dorpat, Estonia (now Tartu). The russification of the university and the expulsion of German faculty, however, led him to move to Moscow University. after graduation in 1897, he continued his education at St. An na Children’s Hospital at the University of Graz, Austria, under Theodor Escherich. Subsequently, he worked at l’Hôpital des Enfantes Malades in Paris with Jacques-Joseph Grancher, Antoine Marfan, and Victor Henri Hutinel. It is interesting to note that both Escherich and Grancher, a close collaborator of Louis Pasteur, are thought to be the first pediatricians for infectious diseases [1].

At the end of 19^th^ century, bacteriologist as a profession, per se, did not exist, but a few scholars, mainly physicians, were engaged in investigations of the microflora of the human gastrointestinal tract and its role in health and disease. This work may mark the beginnings of probiotic and microbiome research. Reminiscent of today, there was no shortage of hype, skepticism and controversies involved.

**Fig. 2:**
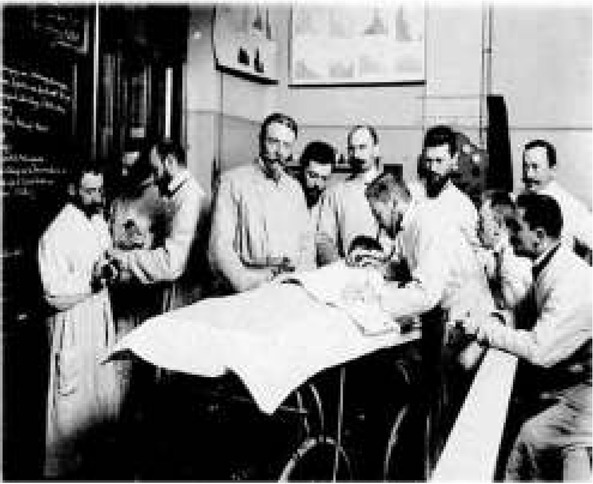
Escherich’s clinic in Graz, Austria. Theodor Escherich (center), Meinhard von Pflauner examining a child, and Józef Brudziński (far left) with Ernst Moro inoculating a laboratory animal.

Theodor Escherich was the discoverer of Bacilllus coli commune, later renamed Escherichia coli in his honor. He is credited with carrying out the first systematic study of intestinal bacteria [2]. Escherich’s clinic attracted researchers not only from Europe, but practically from all over the world (fig. 2). According to one of Escherich’s assistants and later a renowned physician, Bela Schick, the bacterial era was inaugurated in pediatrics by Escherich, who had a longstanding interest in bacterial flora of the gastrointestinal tract [3]. It was believed at the time that bacteria are responsible for decomposition of proteins to harmful putrefaction products that could be absorbed from the bowels into the blood stream, a form of auto-intoxication. Escherich suggested fighting this condition by the introduction of acid-producing bacteria, first described by Louis Pasteur [4], and including carbohydrates to the diet based on a mutual antagonism between the saprophytic intestinal inhabitants and the acid-producing bacteria.

In Escherich’s clinic, Brudziński [5, 6] was to test this hypothesis by performing experiments aimed at com bating intestinal putrefaction in dyspeptic infants using the acid-producing Bacillus lactis aerogenenes (now Enterobacter aerogenes), which was known to ferment sugars with the formation of lactic acid and gas. This organism had previously been found by Escherich to be one of the two dominant bacteria in stools of healthy infants (The other was E. coli. Note that this was before knowing that the dominant intestinal bacteria are strict anaerobes). Brudziński first examined fetid stools of several dyspeptic infants and found that most grew out Proteus vulgaris. He then administered pure cultures of Bacillus lactis aerogenes, which proved successful. The foul smell subsided, and they regained their natural acidic smell. Proteus was now absent. A similar effect was achieved by feeding patients large amounts of milk and sugar. He concluded that the symptoms of auto-intoxication observed in dyspeptic children were due to absorption in the intestine of toxins derived from Proteus. Brudziński also performed experiments with animals. Proteus injected under the skin of mice was lethal, while no symptoms were observed if mixed with food of young dogs and kittens. To identify the source of Proteus found in the stools, Brudziński examined samples of raw and boiled milk for the presence of the bacteria. Proteus grew only in previously boiled milk, seldom in fresh milk, and never in acidic milk.

Research on bacterial microflora of the gastrointestinal tract was quite prolific at the time. Two newly discovered species of intestinal bacteria were added to the list. Ernst Moro discovered a gram-positive bacterium, Bacillus acidophilus (now Lactobacillus acidophilus ), claiming that it is a dominant species in breast-fed infants [7]. At the Pasteur Institute, Henri Tissier observed a Y-shaped bacterium, and, because of its tendency to branch, named it Bacillus bifidus (now Bifidobacterium ) [8]. Tissier disputed Moros’ assertion of the dominance of B. acidophilus, claiming that B. bifidus is of primary importance. The controversy ended with Moro’s own admission that B. bifidus is present in far greater numbers in breast-fed babies, and both agreed that B. acidophilus was predominant in stools of cow’s milk fed babies.

Meanwhile, Elie Metchnikoff , also working at the Pasteur Institute, turned his attention to the newly discovered Bacillus bulgaricus (now Lactobacillus delbruckii subsp. Bulgaricus [9, 10, 11]. This species is not only a powerful acid producer but is also resistant to high acid concentrations. The large numbers of centenarians living in Bulgaria, where lactobacilli-containing yogurt was a dietary staple, convinced Metchnikoff that B. bulgaricus may provide the secret to longevity by replacing the toxin-generating bacteria in the colon. Whether this longevity may be attributed to the health benefits of yogurt or was simply a consequence of unreliable birth records is a matter of contention. Mechnikoff was not a physician and contributed little to experimental work, but was a staunch advocate of the health benefits of sour milk. This caused a media frenzy. The popular French daily, Le Matin, with front page headline: “Vive la Vie!” (Long live Life!), announced that Metchnikoff found an elixir of eternal youth [12]. Subsequent studies with B.bulgaricus in other laboratories put into question whether it can survive in the stomach, much less colonize the colon. Metchnikoff ’s conviction, bordering on obsession, with the notion that acid producing bacteria may prolong life did not go unnoticed. As a result, he has been proclaimed the father of probiotics. His regimen of daily consumption of sour milk probably did not prolong his life by as many years as he had wished for. He died in 1916 at the age of 71. However, his accomplishments in immunology – notably, the discovery of phagocytosis, for which he was awarded the Nobel Prize in 1908 – earned him immortality.

The research exemplified above, relatively high-profile at the time, received recognition in the literature, but was also met with a dose of skepticism, as demonstrated by the following [13]:

“Recently, renewed attention has been called to the possibility of influencing intestinal fermentation and putrefaction by the administration of cultures of bacteria. An old method was to administer brewer’s yeast. More recently Brudziński, Metchnikoff and Tissier have employed cultures of organisms that occasion lactic acid fermentation. The same applies to the administration of sour milk, buttermilk, koumiss, and similar preparations. The value of this form of treatment has not yet been determined. It is doubtful whether the extravagant claims recently put forward will be substantiated by further investigation.”

The concept of influencing the composition of intestinal microflora with the use of mutually antagonistic bacteria was also discussed in terms of individual contributions. Alexander Poehl, a chemist and pharmacist from St. Petersburg was the first to discover that sour milk diminishes intestinal putrefactions [14]. Only later were cultures of lactic-acid producing bacteria used in attempts to sup press this condition. Metchnikoff ’s contribution to researching the topic was questioned in another report [15]:

“Poehl, Brudziński, Fischer, Rovighi and Embden then turned their attention to sour milk and found that that too would inhibit intestinal putrefaction to a certain extent at least. This work is in reality the foundation upon which Metchnikoff built his sour milk therapy. His claim to recognition appears to depend largely on the fact that he popularized this form of administration of lactose and lactic acid”.

Yale researchers Rettger and Cheplin [16] provided a more detailed list of workers in the field with the timeline of their published work:

Poehl (1887) noted that sour milk when ingested decreased intestinal putrefaction. This observation was confirmed by Rovighi (1892), Embden (1894), Brudsiński [sic] (1900) and Fischer (1903). Tissier and Martelly (1902) stated that the chief agent in effecting inhibition of putrefying bacteria is probably the lactic acid produced by the lactic acid bacilli. Tissier and Gasching (1903) found that acid-producing bacilli are able in a sugar-containing medium to arrest the growth of putrefactive organisms, thus confirming the conclusions of Bienstock (1899).

Thus, at the turn of 19^th^ and 20^th^ century, work on the bacterial gastrointestinal microflora thrived. Elie Metchnikoff took notice of Brudzinski’s work. He mentioned it briefly in his philosophical book Études optimistses, translated into English under a provocative title: Prolongation of Life: Optimistic Studies [17]. In addition, in a scientific report, Bacteriotherapie Intestinale [18], Metchnikoff devoted a large section to Brudzinski’s studies, commenting that while the bacteriological studies were marred by the fact that they did not include anaerobic bacteria, this in no way detracted from the importance of the clinical experiments he carried out. He expresses astonishment, that no one followed up on this work. Finally, he concludes that at the end of 19^th^ century, the bases of bacterial therapies had already been established and had given rise to several interesting applications.

This early fascination soon began to wane, but was kept alive for some time, thanks in part to sensational news with the promise of prolonging life, only to die with the advent of Fleming’s “miracle cure” – penicillin. The early pioneers in the field are now largely forgotten. Today, as a consequence of antibiotics overuse and misuse and the growing threat of antibiotic resistant bacteria, we are witnessing the re-emergence of such studies, on a vastly larger scale by a rapid expansion of pro biotic and microbiome research. Technological advances allow us to study human-inhabiting bacteria as a collection of numerous species and to analyze functional, complex interactions between the host and the microbiota, with new hope of preventing and treating a range of human diseases.

As mentioned above, Brudziński, apart from being a physician, was involved in other pursuits such as organization of several hospitals in Poland, launching of the first Polish pediatric journal, Przeglad Pediatryczny (Pediatric Review) in 1908, as well as actively participating in political life. In 1916, a declaration of Emperors Wilhelm II of Germany and Franz Joseph of Austria promised the creation of the Kingdom of Poland. This was regarded as one of main factors in the Polish efforts to regain independence. On November 6^th^, 1916, under the headline: “Warsaw cheers promised freedom”, The New York Times reported [19]:

*“The ceremony was short and simple. Precisely at noon, General Besseler, wearing the decorations granted for the reduction of Antwerp and the Polish fortresses mounted the dais of the gala ballroom of the old Jagiellonian Castle, and in the name of Germany’s sovereign read the imperial manifesto in ringing, soldiery tones... President Brudziński of the recently elected City Council, who is rector of the University of Warsaw, advanced before the dais and in the Polish tongue gave thanks to the imperial decree... President Brudziński, who was in plain civilian attire, without decorations, seemed to represent the spirit not of the ancient Poland and the Polish chivalry but of modern intellectual Poland.”*

The scientific careers of Brudziński and Moro followed a remarkably parallel path; aside from their bacteriological work under Escherich, both discovered neurological signs, now bearing their names. The Moro reflex in infants is a response to sudden loss of support and is believed to be the only unlearned fear in humans. Brudziński’s career ended with his premature death, while the career of Moro, whose wife was of Jewish origin, was cut short by the Nazi rule.
